# A biophysically realistic computer model of Alzheimer pathology to guide the development of symptomatic drugs

**DOI:** 10.1186/1471-2202-13-S1-P133

**Published:** 2012-07-16

**Authors:** Patrick D Roberts, Athan Spiros, Hugo Geerts

**Affiliations:** 1 In Silico Biosciences, Inc., Lexington, MA 02421, USA; 2 Department of Biomedical Engineering, Oregon Health & Science University, Portland, OR 97239. USA

## 

The pharmaceutical industry is approaching unsustainable research costs to develop new drug therapies for mental disease because of the high failure rate in clinical trials. These failures are due to limitations of pre-clinical studies in animal models that fail to predict the efficacy of new drugs in human subjects. The gap between pre-clinical trials and clinical trials is particularly difficult in complex mental diseases such as Alzheimer’s disease because of the complex dynamics of the brain and the multiple chemical pathways that drugs can affect.

However, many biological mechanisms associated with Alzheimer’s disease are now understood, and computational power and methods have reached the point for practical modeling of pathologies of Alzheimer’s disease. Numerical models can combine the information from animal studies of brain circuitry with data from human clinical trials of drug actions. Furthermore, complex interactions of multiple receptor targets can be predicted by a biophysical model of brain function.

We introduce numerical models of neuronal microcircuitry that are associated with symptoms of Alzheimer’s disease. The emphasis is on the dynamics of these neural systems and how their dynamics are modified by therapeutic drugs. Unlike the current state-of-the-art methods of estimating therapeutic efficacy, the computational platform yields a significant increase in the predictive correlation with data from clinical trials.

To develop a biophysical model for functional cognitive performance, we implemented a conductance-based computer model of multicompartment neuronal cell types in a cortical brain circuit for working memory using preclinical data on receptor pharmacology of catecholamine and cholinergic neurotransmitters. The pathology of Alzheimer’s disease was subsequently implemented as a loss of synapses and neurons and decreased cholinergic tone and the model as further calibrated the model with clinical ADAS-Cog results on acetylcholinesterase inhibitors and 5-HT6 antagonists. As an independent validation, we reproduce clinical data showing that ApoE genotype, implemented as a specific synapse loss and a decline in cholinergic tone, reduces the network performance much more in mild cognitive impairment conditions than at later stages of Alzheimer’s disease pathology.

As a further example, we explore the reason for the differential effect of memantine, an NMDA subunit selective weak inhibitor in early and late Alzheimer’s disease pathology. The preferential inhibition of the NMDA-NR2C/NR2D subunits that are located on inhibitory interneurons compensates for the greater excitatory decline observed with stronger pathology.

This quantitative systems pharmacology mechanistic disease model uses the full human pharmacology for clinical candidates in a humanized calibrated model of Alzheimer’s disease. Complementary to traditional animal models, the model has potential to assess the potential off-target effects of disease-modifying drugs, the consequences of pharmacologically active unique human metabolites, the effect of comedications and the impact of a small number of well-described genotypes.

